# Malaria Incidence, Growth, and their Relationship among a Cohort of Malawian Children

**DOI:** 10.4269/ajtmh.25-0568

**Published:** 2026-07-07

**Authors:** Divya Hosangadi, Liana R. Andronescu, Patricia Mawindo, Don P. Mathanga, Terrie Taylor, Miriam K. Laufer, Andrea G. Buchwald

**Affiliations:** ^1^ Department of Epidemiology and Public Health, University of Maryland, Baltimore School of Medicine, Baltimore, Maryland, USA;; ^2^ Center for Vaccine Development and Global Health, University of Maryland, Baltimore School of Medicine, Baltimore, Maryland, USA;; ^3^ Blantyre Malaria Project, Kamuzu University of Health Sciences, Blantyre, Malawi;; ^4^ Department of Osteopathic Medicine, Michigan State University, East Lansing, Michigan, USA;; ^5^ Department of Clinical Sciences, Liverpool School of Tropical Medicine, Liverpool, United Kingdom

## Abstract

Child growth impairment and *Plasmodium falciparum* (*P. falciparum*) infection pose substantive public health challenges, particularly in sub-Saharan Africa. There is a paucity of evidence regarding how *P. falciparum* infection, which causes clinical malaria, relates to child growth in children under 24 months old. To assess the temporal associations between *P. falciparum* infection and weight-for-age Z score (WAZ), a secondary analysis of a prospective cohort study conducted from 2016 to 2018 in which 86 Malawian children (*n* = 649 observations) were followed from birth to 24 months of age was conducted. The incidence of *P. falciparum* infection was assessed, as well as whether *P. falciparum* infection was associated with subsequent reductions in WAZ and whether lower WAZ scores were associated with increased risk of subsequent *P. falciparum* infection. The authors also investigated whether being mildly to moderately underweight (WAZ < –1.0) at birth was associated with a shorter time to the first *P. falciparum* infection. *Plasmodium falciparum* infection (symptomatic or clinical malaria), asymptomatic *P. falciparum* infection, and clinical malaria were identified separately in all analyses. More than 30% of children experienced *P. falciparum* infection within the first 6 months of life. Being mildly to moderately underweight was associated with a significantly higher incidence of *P. falciparum* infection in the subsequent 3 months (adjusted incidence rate ratio: 1.25 [1.02–1.52]). Being mildly to moderately underweight at birth was associated with a shorter time to the first asymptomatic *P. falciparum* infection. The study findings reveal a high burden of *P. falciparum* infection among children <6 months old and highlight the need for targeted interventions to address low WAZ in children as an approach to potentially reducing subsequent malaria risk.

## INTRODUCTION

Children in sub-Saharan Africa (SSA) disproportionately experience a combined high burden of malnutrition and *Plasmodium falciparum* (*P. falciparum*) infection, which can cause clinical malaria.^1^^,^^2^ The first malaria vaccine (RTS,S) was prequalified by the WHO in 2022, followed by the R21 vaccine in 2023.^3^^,^^4^ These vaccines have only recently been introduced into routine care across malaria-endemic countries in SSA,^5^ and the global clinical malaria burden remains high. An estimated 282 million cases and 610,000 malaria deaths occurred in 2024 alone,^6^ of which more than 6 million cases and 16,000 deaths were estimated in Malawi.^7^ Children under 5 years old experience the greatest burden of severe malaria illness,^8^ accounting for ∼75% of malaria deaths.^9^ Likewise, childhood malnutrition, which can manifest as underweight, wasting, or stunting, contributes to more than 3 million deaths among children under 5 years of age annually.^1^ Approximately 16% to 21% of children under 5 years of age in SSA and between 12% and 18% in Malawi are underweight,^10^^–^^16^ defined as having a weight-for-age Z-score (WAZ) at least 2 SDs below child growth standards established by the WHO.^17^ *Plasmodium falciparum* infection, regardless of whether it results in clinical malaria, and child malnutrition have been associated with downstream adverse health outcomes, including increased risk of all-cause mortality,^13^^,^^18^^,^^19^ subsequent growth impairment,^20^^,^^21^ and cognitive impairment,^13^^,^^22^^–^^27^ highlighting the importance of addressing both health issues.

To adequately address the underlying causes of child malnutrition and malaria, and reduce the burden of these two conditions, there is a need to characterize the directional relationship between malaria and growth impairment. The overlapping burden and risk factors for malaria and growth impairment, including socioeconomic factors,^28^^–^^32^ environmental and seasonal factors,^33^ and past or concurrent parasitic infections,^14^^,^^20^^,^^28^^,^^30^^,^^34^^–^^38^ add to the complexity of understanding their relationship. Although challenging, disentangling the temporal relationship between *P. falciparum* infection and child growth impairment using longitudinal data is a crucial first step to informing causal inference assessments and shaping the implementation of effective interventions targeting both conditions.

Past evidence regarding the associations between malaria and growth impairment has been mixed.^1^^,^^39^ Studies on malaria and growth impairment have frequently been cross-sectional and thus unable to establish temporality.^1^^,^^40^^–^^44^ Longitudinal studies have generally assessed whether growth impairment was a risk factor for subsequent malaria and have yielded mixed results.^20^^,^^39^^,^^45^ Some authors reported that growth impairment, including being underweight, increased the risk of subsequent malaria,^45^^,^^46^ indicating that interventions targeting growth impairment could help reduce subsequent malaria risk. Others, however, found no association when assessing weight over time.^47^ Additionally, there is evidence that *P. falciparum* infection is associated with increased risk of subsequent stunting and wasting,^20^^,^^48^^,^^49^ suggesting that preventing *P. falciparum* infection can improve child growth.^50^^,^^51^ Studies in which the impact of *P. falciparum* on infant weight was assessed, however, are either less common or revealed no significant association.^2^ There is a lack of studies in which the bidirectional associations between malaria and growth have been characterized within the same population.

Assessing the relationship between these conditions among children is particularly challenging because of the lack of preexisting *P. falciparum* data in this age group, particularly among those under 6 months old.^1^^,^^49^^,^^52^ Children under 2 years old are susceptible to both asymptomatic *P. falciparum* infection^53^^–^^55^ and clinical malaria;^56^^,^^57^ however, *P. falciparum* burden in children under 6 months old has been understudied because of the assumption that this age group has protective maternal antibodies and therefore low malaria incidence.^52^^,^^58^ Past evidence regarding malaria in children is often focused on children over 6 months old^49^^,^^53^^,^^59^^,^^60^ or those within broadly aggregate age ranges (e.g., all children under 5 years old).^9^^,^^61^ Likewise, although some studies have been conducted to assess growth impairment and asymptomatic *P. falciparum* infection,^20^^,^^44^ fewer have been conducted to evaluate the association between asymptomatic infection and WAZ.^49^ Studying asymptomatic *P. falciparum* infection remains important, however, because children under 5 years of age in endemic regions have elevated risk for prolonged subclinical undetected infections,^61^^,^^62^ which have been linked to elevated risk for anemia,^61^ cognitive impairment, and certain forms of growth impairment.^20^^,^^55^^,^^62^

The present study was conducted to assess the incidence of both clinical malaria and asymptomatic *P. falciparum* infection and their relationship to WAZ among children from birth to 2 years of age. An analysis of longitudinal data collected from a prospective cohort study in a high-malaria-burden setting in Malawi is reported to disentangle the temporal relationship between malaria and growth. The incidence of *P. falciparum* infections over time was assessed, as well as whether *P. falciparum* infection was associated with subsequent changes in WAZ, whether having a low WAZ was associated with subsequent risk of *P. falciparum* infection, and whether a low birth WAZ was associated with differences in time to experiencing a child’s first *P. falciparum* infection. The aim for the present analysis is to further clarify the nature of the relationship between malaria and growth during a critical age window for interventions to reduce both long-term growth impairment and malaria incidence.

## MATERIALS AND METHODS

### Study population.

This is a secondary analysis of data from a single-center prospective cohort study^54^ conducted between January 2016 and November 2018 at the Mfera Clinic, located in Chikwawa, Malawi, an area of high malaria transmission.^63^ Participants were children of mothers who attended the Mfera antenatal clinic or well-child clinic.

### Inclusion and exclusion criteria.

Children were included in the cohort if the parent or guardian consented to the child’s participation, the child was between 0 and 3 months old at enrollment, the mother was HIV-negative, the mother planned to reside in the study locality for 2 years, and the mother was willing to adhere to study protocols and regularly attend clinic visits.

Children were excluded at enrollment if they 1) had an acute illness that required hospitalization, 2) had symptoms of severe malaria, 3) had symptoms of moderate to severe anemia, or 4) regularly took a medication that had antimalarial properties.

### Data collection.

All children included in the present analysis were enrolled at birth, when data on child characteristics and maternal pregnancy history were collected. Blood samples were collected from cord blood and placental samples at delivery to test for maternal *P. falciparum* infection during pregnancy. Children were scheduled to attend the Mfera clinic for quarterly visits until 24 months of age, and mothers were encouraged to bring their children to the clinic for all episodes of illness. Except for two observations, all children’s quarterly visits occurred within 14 days of the intended age. Data on weight, temperature, malaria symptoms, and other measures were collected at each visit. At all scheduled visits, as well as any unscheduled visits in which children presented with malaria symptoms, blood samples were collected for filter paper dried blood spot assays, malaria rapid diagnostic tests (RDTs), and malaria smears. Dried blood spots were collected regardless of malaria symptoms. Filter paper dried blood spots were shipped to the University of Maryland, Baltimore, for quantitative polymerase chain reaction (qPCR) testing to detect the *P. falciparum* 18s ribosomal RNA gene.^64^

### Exposure and outcome definitions.

#### Malaria.

Clinical malaria was defined as having a positive RDT result paired with any of the following symptoms: a current fever (>37.5°C); a fever within the previous 48 hours; or headache, malaise, vomiting, or weakness at the time of presentation. Children who met the case definition were treated on site with a standard course of artemether–lumefantrine. Asymptomatic *P. falciparum* infection was defined as having a positive qPCR test result but not meeting the clinical case definition. Because of the delay between sample collection and qPCR testing, asymptomatic infections were not treated. These two types of infections (clinical malaria and asymptomatic *P. falciparum* infection) were analyzed separately and together as *P. falciparum* infection. Asymptomatic *P. falciparum* infection, clinical malaria, and *P. falciparum* infection were assessed as three separate measures in three separate models, each corresponding to an overarching research objective, as described in the Regression Analyses section. Any *P. falciparum* infection episode that occurred within 2 weeks of the previous episode was treated as a single event. In this case, if the episodes were either both asymptomatic *P. falciparum* infections or both clinical malaria cases, only the date of the first visit was counted as the date of the episode. If, however, one visit involved clinical malaria and the other involved an asymptomatic *P. falciparum* infection, only the clinical malaria visit was counted in the analysis, regardless of which visit occurred first. Maternal malaria refers to maternal *P. falciparum* infection at delivery or placental malaria based on positive qPCR test results from dried blood spots of cord or placental blood.

#### Growth.

The authors focused on WAZ to assess child growth because of missing data on child length. The WAZ was determined on the basis of the WHO 2006 Child Growth Standards^17^ and calculated using the WHO Anthro Survey Analyser software (WHO; Geneva, Switzerland), which calculates the value on the basis of child age, weight, and sex.^17^^,^^65^

#### Covariates.

**Figure 1.**
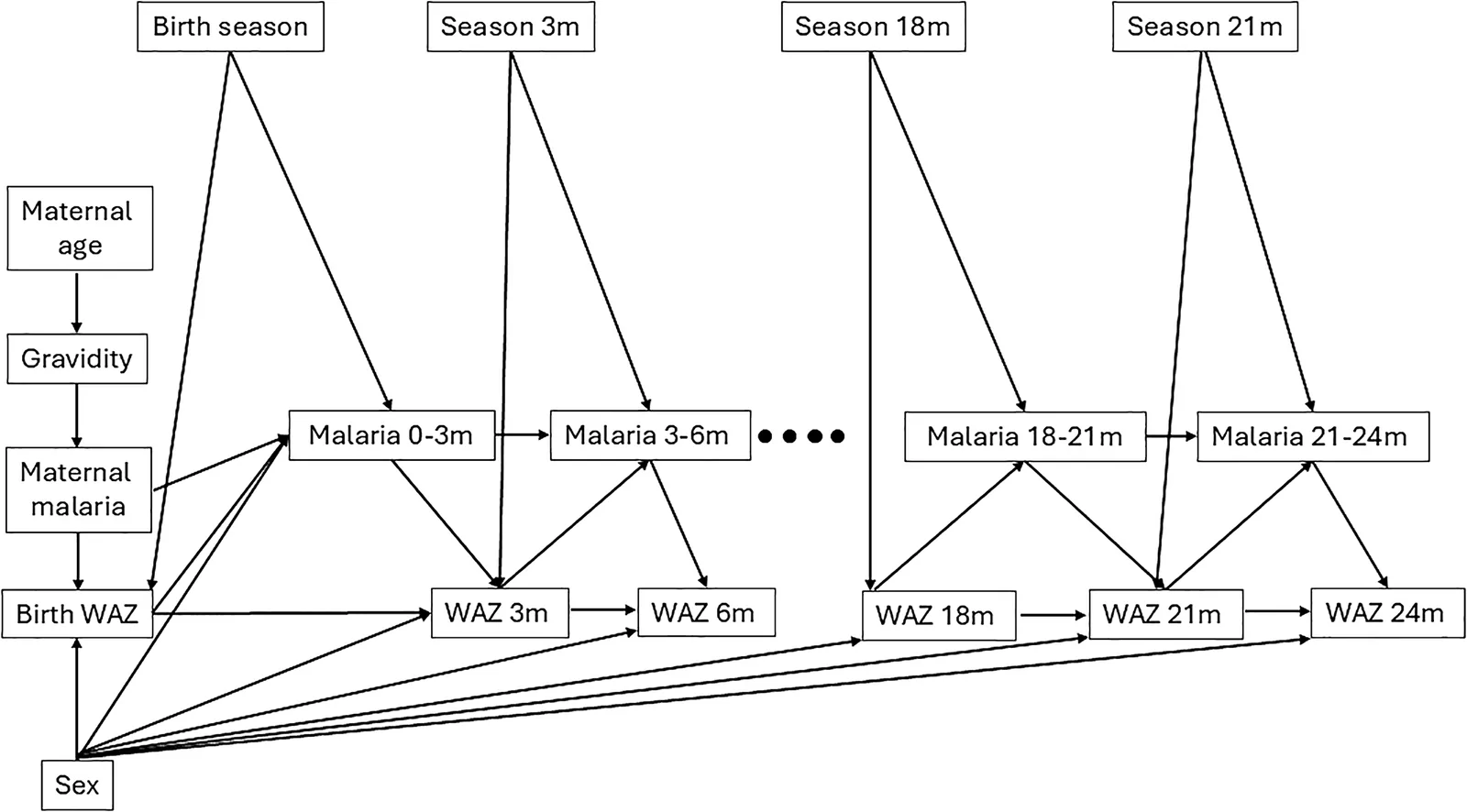
Directed acyclic graph (DAG) revealing the hypothesized relationship among malaria, weight-for-age Z-score (WAZ), and assessed covariates. The DAG was developed a priori on the basis of a review of the peer-reviewed literature and consultation with subject matter experts. Unmeasured confounders that are not shown in the DAG and that could not be accounted for in the analysis include but are not limited to socioeconomic status, child length, and diet, although mothers were advised to exclusively breastfeed for the duration of the study. M = month.

Maternal age (categorized as <20 years, 20 to <25 years, and ≥25 years), gravidity (primigravid versus multigravida), maternal malaria, birth WAZ, child sex, whether the birth was during the rainy season, whether the quarterly visit occurred during the rainy season (December to March), *P. falciparum* infection status in the previous time period, and a WAZ less than –1.0 in the previous time period were considered as potential covariates in regression models. Covariates were selected for inclusion in multivariable regression models using a directed acyclic graph developed on the basis of a literature review and subject matter expertise (Figure 1).

## STATISTICAL ANALYSES

Descriptive exploratory analyses were conducted, and two analytical objectives were evaluated using regression (objectives 1 and 2) and survival analyses (objective 2). Most statistical analyses were conducted using SAS Version 9.4 (SAS Institute, Cary, NC). The survival analyses and generation of certain figures were performed in R Version 4.4.2 (R Foundation, Vienna, Austria).

### Exploratory analysis.

A univariate analysis was conducted to examine the distribution of covariates at enrollment among all children and stratified by whether the child experienced multiple *P. falciparum* infection episodes by the end of follow-up. The χ^2^ or Fisher’s exact test was used to determine if children who experienced multiple *P. falciparum* infection episodes exhibited significantly different distributions of baseline characteristics. Additionally, the percentages of children in each time period who experienced at least one episode of *P. falciparum* infection, asymptomatic *P. falciparum* infection, and clinical malaria were plotted. The authors also estimated, for each *P. falciparum* infection outcome, the mean total number of episodes experienced by study end, the total number of episodes across all children, the incidence rates per person-year, and the number of participants who experienced at least one episode by study end. Mean WAZ scores and 95% CIs (assuming a t-distribution) for each quarterly period were plotted, stratified by sex and by whether the child experienced more than four *P. falciparum* infection episodes by the end of the follow-up period.

### Regression analyses.

Regression analysis was used to examine objectives 1 and 2, described below. For each objective, all *P. falciparum* infections were evaluated as *P. falciparum* infection, asymptomatic *P. falciparum* infection, and clinical malaria in three separate models.

***Objective 1:*** To examine the association between *P. falciparum* infection and subsequent WAZ.

The exposure variables were *P. falciparum* infection measures, defined as experiencing at least one episode of *P. falciparum* infection, asymptomatic *P. falciparum* infection, or clinical malaria during the quarterly period, depending on the model. The outcome variable was WAZ, measured as the change in WAZ from the beginning to end of each quarterly period (ΔWAZ). The regression models indicated whether the mean ΔWAZ over the quarterly period differed significantly between individuals with a malaria outcome and those without any *P. falciparum* infection. A negative value for mean ΔWAZ indicates that, on average, WAZ declined during each quarterly period.

Mixed-effects linear regression accounting for repeated measures for each participant was conducted on the basis of each quarterly visit, assuming a heterogeneous Toeplitz covariance matrix. To ensure temporality of malaria exposure and WAZ outcome measures, malaria exposure episodes diagnosed on the same day of a quarterly visit were assumed to affect only WAZ measured at the next quarterly visit. Multivariable models were adjusted for child sex and whether the WAZ was less than –1.0 (considered mildly to moderately underweight) at the beginning of the quarterly time period.

***Objective 2:*** To examine the association between WAZ and subsequent *P. falciparum* infection.

The exposure variable was defined as mildly to moderately underweight.^66^ There were too few children with WAZ values less than –2.0 to assess that measure. The *P. falciparum* infection measure was defined as the outcome variable, examining whether the incidence rates of *P. falciparum* infection, asymptomatic *P. falciparum* infection, or clinical malaria episodes during the quarterly time period differed by whether the child was mildly to moderately underweight at the beginning of the time period. Negative binomial regression with a Toeplitz covariance matrix was used to account for random effects from repeated measures. Adjusted incidence rate ratios (aIRRs) controlled for child sex, whether it was the rainy season at the beginning of the time period, and whether the child experienced any *P. falciparum* infection episodes in the previous time period (Figure 1).

### Survival analysis.

As part of assessing objective 2, Kaplan–Meier curves of time to first *P. falciparum* infection episode were evaluated to determine if birth WAZ was associated with time to experiencing first episodes of *P. falciparum* infection, asymptomatic *P. falciparum* infection, or clinical malaria.

The exposure variable was defined as whether the child was mildly to moderately underweight at birth.^66^ Underweight (WAZ < –2.0) and low birth weight (birth weight <2,500 g) were initially considered, but too few children had these characteristics. The outcome variables were defined as time to first episodes of *P. falciparum* infection, asymptomatic *P. falciparum* infection, and clinical malaria. The proportional hazards assumption was tested using Schoenfeld residuals. Kaplan–Meier curves were used to assess whether the time to experiencing the first episode of *P. falciparum* infection differed significantly between children who were mildly to moderately underweight at birth and those with a birth WAZ ≥ –1.0. *P*-values were calculated using the Gehan–Breslow method.

## RESULTS

### Study population characteristics.

For the study analyses, 86 of the 108 children (79.63%) enrolled in the original Mfera cohort study were included; 22 children were excluded because of missing data on baseline covariates. All children were enrolled at birth. There were 649 quarterly observations, and children contributed a mean of 7.5 of 8 possible quarterly observations, with a mean follow-up time of 1.87 years (95% CI: 1.81–1.94 years). Baseline variables included child sex, birth weight, birth season, maternal malaria, primigravidity, and maternal age (Figure 1). Most children were born during the dry season (75.58%) and were not their mother’s first pregnancy (82.56%), whereas 25.58% were born to mothers who experienced maternal malaria (Table 1). Baseline characteristics did not differ by whether the child experienced multiple *P. falciparum* infection episodes by study end (Table 1). The mean WAZ remained negative from birth onward and generally declined over time, regardless of whether the child experienced more than four *P. falciparum* infection episodes by study end (Supplemental Figures 1 and 2).

**Table 1:** Distribution of characteristics at baseline among children (*N* = 86)

Characteristic	All (*N* = 86), *n* (%)	With <2 *P. falciparum* Infection Episodes by End (*n* = 21), *n* (%)	Multiple *P. falciparum* Infection Episodes by End (*n* = 65), *n* (%)	*P*-Value
Sex				
Male	37 (43.02)	6 (28.57)	31 (47.69)	0.14
Female	49 (56.98)	15 (71.43)	34 (52.31)	
Low birthweight (<2,500 g)				
Yes	7 (8.14)	3 (14.29)	4 (6.15)	0.35
No	79 (91.86)	18 (85.71)	61 (93.85)	
Born during rainy season				
Yes	21 (24.42)	4 (19.05)	17 (26.15)	0.57
No	65 (75.58)	17 (80.95)	48 (73.85)	
Maternal malaria				
Yes	22 (25.58)	5 (23.81)	17 (26.15)	1.00
No	64 (74.42)	16 (76.19)	48 (73.85)	
Primigravid				
Yes	15 (17.44)	2 (9.52)	13 (20.00)	0.34
No	71 (82.56)	19 (90.48)	52 (80.00)	
Maternal age (years)				
<20	15 (17.44)	1 (4.76)	14 (21.54)	0.21
20 to <25	26 (30.23)	7 (33.33)	19 (29.23)	
≥25	45 (52.33)	13 (61.90)	32 (49.23)	

*P. falciparum* = *Plasmodium falciparum.*

Percentage distribution of characteristics at birth among children participating in the study observations across all time points. Baseline characteristics are reported for all children and stratified by whether the child experienced multiple *P. falciparum* infection episodes by study end. *P. falciparum* infection includes both asymptomatic *P. falciparum* infection and clinical malaria. The rainy season is considered to occur between December and March. Maternal malaria is defined as having maternal *P. falciparum* infection at delivery or placental malaria.

### Malaria incidence.

**Figure 2.**
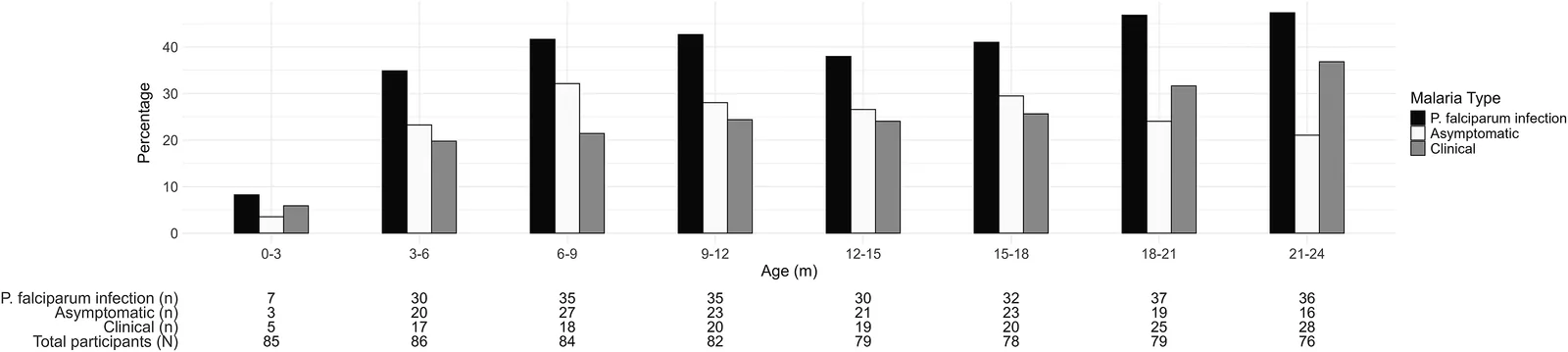
Percentage of children who experienced at least one episode of *Plasmodium falciparum* (*P. falciparum*) infection, asymptomatic *P. falciparum* infection, or clinical malaria by age in months. The numerator of the percentage represents the number of children who had at least one episode during the quarterly period. The denominator is the total number of children present in the study during that quarterly period. The numbers for the numerator and denominator are printed below the figure. *P. falciparum* infections included both asymptomatic *P. falciparum* infection and clinical malaria.

The percentage of children within each time period who experienced ≥1 episode of *P. falciparum* infection was lowest at ages 0 to 3 months (8.23%), then increased and remained above 30% from ages 3 months and older (Figure 2). More than 10% of children experienced multiple *P. falciparum* infection episodes between the ages of 3 and 6 months (Supplemental Figure 3). Children experienced a mean total of 4.36 *P. falciparum* infection episodes with a total of 375 episodes by study end, equating to an incidence of 2.32 *P. falciparum* infection episodes per person-year (Table 2). The incidence per person-year of asymptomatic *P. falciparum* infection (1.04 episodes) was similar to that of clinical malaria (1.28 episodes). By study end, 77 (89.53%) children experienced at least one episode of *P. falciparum* infection (Table 2).

**Table 2:** Frequency and incidence of *Plasmodium falciparum* infection, asymptomatic *Plasmodium falciparum* infection, and clinical malaria episodes within the first 24 months of life among a cohort of Malawian children

Characteristic	*P. falciparum* Infection	Asymptomatic *P. falciparum* Infection	Clinical Malaria
Mean number of episodes per child by the end of the follow-up period (95% CI)	4.36 (3.62–5.09)	1.95 (1.58–2.33)	2.41 (1.93–2.88)
Total number of episodes by the end of the follow-up period	375	168	207
Incidence rate per person-year (95% CI)	2.32 (2.10–2.58)	1.04 (0.90–1.21)	1.28 (1.12–1.47)
Number of participants experiencing at least one episode by the end of the follow-up period (*N* = 86)	77 (89.53)	69 (80.23)	64 (74.42)

*P. falciparum* = *Plasmodium falciparum.*

Incidence rates were calculated using Poisson regression to model the cumulative number of episodes of each type of infection experienced by the participant by the end of follow-up, with an offset of log person-years. Among participants who experienced at least one episode during the study, 51.95% (*n* = 40) experienced asymptomatic *P. falciparum* infection as their first episode. *Plasmodium falciparum* infection includes both asymptomatic *P. falciparum* infection and clinical malaria.

#### Objective 1: Association of P. falciparum infection with subsequent WAZ.

The mean ΔWAZ from the beginning to the end of each quarterly period was negative among both children with *P. falciparum* infection (ΔWAZ: –0.01 [–0.11 to 0.08]) and those without *P. falciparum* infection (ΔWAZ: –0.1 [–0.18 to –0.02]) during the time period, indicating that WAZ declined regardless of whether the child had ≥1 *P. falciparum* infection episode during that time period (Table 3). Similar results were observed among children with asymptomatic *P. falciparum* infection (ΔWAZ: –0.02 [–0.16 to 0.12]), whereas no change in WAZ (ΔWAZ: 0.00 [–0.15 to 0.15]) was observed among children who experienced clinical malaria during the time period. Children who experienced no *P. falciparum* infection during the time period exhibited significantly larger declines in WAZ than those who had experienced *P. falciparum* infection (*P* = 0.03) or clinical malaria (*P* = 0.03), but the absolute magnitudes of these differences were small (Table 3).

**Table 3:** Association between experiencing at least one episode of *Plasmodium falciparum* infection, asymptomatic *Plasmodium falciparum* infection, or clinical malaria and subsequent change in weight-for-age Z-score using linear mixed-effects models (*N* = 86; observations = 649)

Malaria Exposure	Participant Infection Status During Time Period	Number of Observations (*n* = Time Periods)	Mean Decline in WAZ from the Beginning to the End of the Time Period (ΔWAZ)	*P*-Value Comparing ΔWAZ between Observations with Versus without *P. falciparum* Infection Exposure
None	No episodes	407	−0.10 (–0.18 to –0.02)	Ref.
*P. falciparum* infection	≥1 episode	242	−0.01 (–0.11 to 0.08)	0.03
Asymptomatic	≥1 episode	90	−0.02 (–0.16 to 0.12)	0.20
Clinical	≥1 episode	90	0.00 (–0.15 to 0.15)	0.03

*P. falciparum* = *Plasmodium falciparum*; Ref. = reference; WAZ = weight-for-age Z-score; ΔWAZ = the change in WAZ from the beginning to the end of a quarterly period.

A negative value for ΔWAZ indicates that WAZ declined from the beginning to the end of the quarterly time period. A significant *P*-value (*P* <0.05) indicates that the degree of decline in WAZ between observations experiencing *P. falciparum* infection and observations with no *P. falciparum* infection during the time period significantly differed. *P*-values obtained from multivariable linear mixed effects models adjusted for sex and WAZ at a previous time point. *Plasmodium falciparum* infection includes both asymptomatic *P. falciparum* infection and clinical malaria.

#### Objective 2: Association of WAZ with subsequent P. falciparum infection.

**Figure 3.**
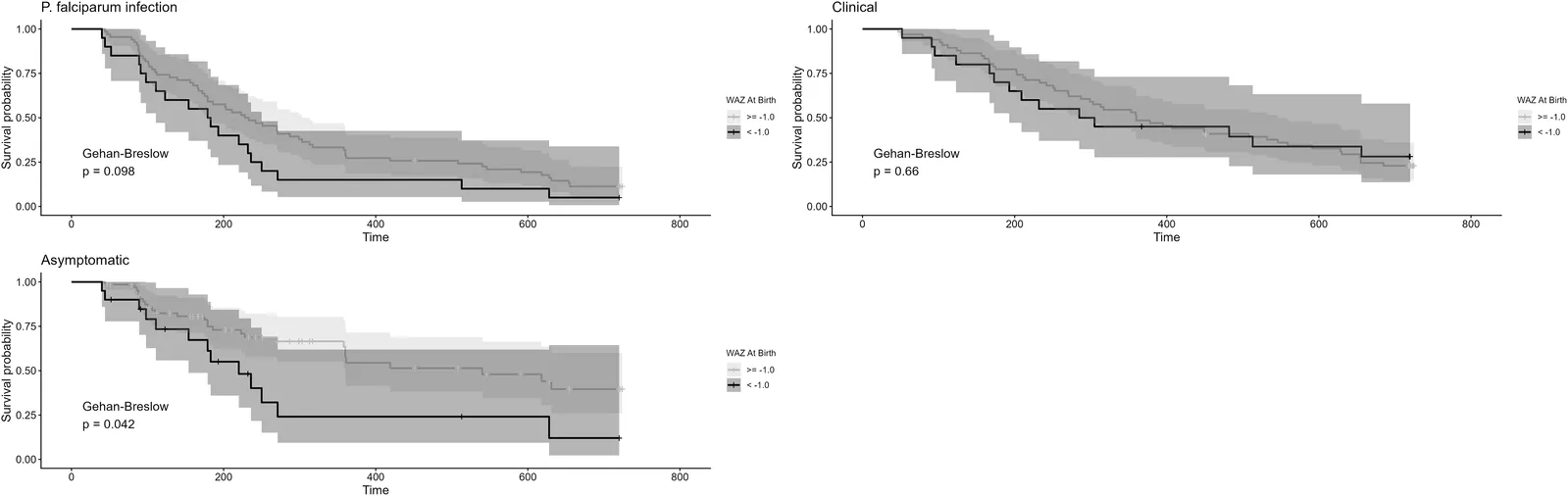
Survival curves representing the time (in days) to the first *Plasmodium falciparum* (*P. falciparum*) infection, asymptomatic *P. falciparum* infection, and clinical malaria event among participants with a birth weight-for-age Z-score (WAZ) < –1.0 (black) versus those with a birth WAZ ≥ –1.0 (gray). The time in days from birth to first *P. falciparum* infection (top left), asymptomatic *P. falciparum* infection (bottom left), and clinical malaria (right) was measured among participants who were mildly to moderately underweight at birth and those with a birth WAZ ≥ –1.0. Mildly to moderately underweight was defined as a birth WAZ < –1.0. Participants who were mildly to moderately underweight had a shorter time to their first asymptomatic *P. falciparum* infection event. Time is defined in days since birth. *Plasmodium falciparum* infection includes both asymptomatic *P. falciparum* infection and clinical malaria.

The authors found that children who were mildly to moderately underweight at the beginning of a quarterly time period had a significantly higher incidence of *P. falciparum* infection (aIRR: 1.25 [1.02–1.52]; *P* = 0.03) by the end of that time period (Table 4). Results were similar but not statistically significant when assessing the incidence of clinical malaria (aIRR: 1.33 [0.99–1.78]; *P* = 0.06; Table 4). Additionally, the authors found that being mildly to moderately underweight at birth was associated with a significantly shorter time to the child’s first episode of asymptomatic *P. falciparum* infection (*P* = 0.04), but not with significantly shorter time to their first *P. falciparum* infection or first clinical malaria episode (Figure 3).

**Table 4:** Association between mildly to moderately underweight status at the beginning of a quarterly period and experiencing at least one episode of any *Plasmodium falciparum* infection, asymptomatic *Plasmodium falciparum* infection, and clinical malaria within the following 3 months, using negative binomial regression

WAZ Exposure	Model Description	IRR of *P. falciparum* Infection Outcome in Following 3 Months (IRR [95% CI])					
		*P. falciparum* Infection	*P*	Asymptomatic Infection	*P*	Clinical Malaria	*P*
Mildly to moderately underweight (ref: WAZ ≥ –1.0)	Unadjusted	1.28 (1.0–1.63)	0.05	1.12 (0.74–1.70)	0.60	1.39 (1.0–1.95)	0.05
	Adjusted	1.25 (1.02–1.52)	0.03	1.13 (0.77–1.64)	0.54	1.33 (0.99–1.78)	0.06

IRR = incidence rate ratio; *P. falciparum* = *Plasmodium falciparum*; WAZ = weight-for-age Z-score.

There were 649 observations used in the analysis, corresponding to 86 participants who were measured from birth to 24 months. All adjusted models controlled for child sex, rainy season at the start of the time period, and whether the participant experienced malaria in the previous time period. The model compared incidence rates of *P. falciparum* infection outcomes over the quarterly period among participants who were mildly to moderately underweight versus those with a WAZ ≥ –1.0 at the beginning of the time period. The model accounted for repeated measures within participants and featured a Toeplitz covariance structure. Mildly to moderately underweight is defined as a WAZ < –1.0. *Plasmodium falciparum* infection includes both asymptomatic *P. falciparum* infection and clinical malaria.

## DISCUSSION

Using longitudinal data from a closely followed cohort of children enrolled at birth in rural southern Malawi, the temporality of the short-term relationships between *P. falciparum* infection and WAZ was assessed. The authors found a high rate of both asymptomatic *P. falciparum* infection and clinical malaria in infancy. Being mildly to moderately underweight was associated with a significantly higher incidence of *P. falciparum* infection, and children born mildly to moderately underweight experienced asymptomatic *P. falciparum* infection significantly earlier than those with higher birth WAZ values. Children exhibited decreasing mean WAZ values throughout the study, regardless of the number of malaria episodes the child experienced by the end of the follow-up period. The authors found no evidence that *P. falciparum* infection or clinical malaria, compared with no *P. falciparum* infection, were associated with greater reductions in subsequent WAZ. In fact, children with no *P. falciparum* infection during a time period unexpectedly exhibited significantly larger reductions in WAZ, although these trends were small in absolute magnitude and unlikely to be clinically relevant.

Incidences of both asymptomatic *P. falciparum* infections and clinical malaria were high throughout the study period, including among children under 6 months old. More than 30% of children experienced either asymptomatic *P. falciparum* infection or clinical malaria by 6 months of age. These data, as previously shown, reveal evidence of waning immunity after 3 months of age.^67^ However, contrary to the previously cited understanding of malaria epidemiology in young children,^52^^,^^58^^,^^68^^,^^69^ infections before 3 months of age indicate that passively acquired maternal antibodies may be insufficient to protect against early-life *P. falciparum* clinical malaria, particularly in regions with a high malaria burden. The high incidence of asymptomatic *P. falciparum* infections is particularly important to note, as such infections may not prompt a protective response against future infections by new genotypes.^55^ On average, approximately half of the episodes that children experienced in their first 2 years of life were asymptomatic infections, and by the end of the follow-up period, nearly 90% of children experienced at least one *P. falciparum* infection episode.

There is a relatively limited number of recent studies on longitudinal relationships between *P. falciparum* infection and WAZ.^20^^,^^39^^,^^45^^,^^49^^,^^54^ The present study’s findings contrast with a previous analysis^54^ on data from multiple Malawian cohorts, including the present study’s population, which revealed that children who experienced malaria before 6 months old had significantly lower WAZ scores from 6 to 24 months of age. This suggests that early-life *P. falciparum* infection may have a longer-term association with WAZ reductions, despite the lack of an observed clinically relevant short-term (within 3 months) association. A recent study also revealed that children 6 to 18 months of age with asymptomatic *P. falciparum* infection had lower WAZ values overall but revealed no significant association at most time points when each quarterly age was assessed separately, except at 12 months of age.^49^ Other cohort studies on the association between malaria and subsequent child growth have often been focused on child length or height,^20^^,^^70^^,^^71^ primarily older age groups,^20^^,^^45^^,^^71^^–^^73^ or *P. vivax* infections,^73^ thus making comparisons to the present analysis difficult. The lack of a clinically relevant association between *P. falciparum* infection and subsequent reductions in WAZ found here may be a result of the overall poor health and high rate of *P. falciparum* infection in the cohort; however, the high participant retention and frequent clinic visits of the study population likely indicate that the present study’s cohort is healthier than average for the setting.

The study findings that being mildly to moderately underweight is associated with both an elevated incidence of *P. falciparum* infection within the subsequent 3 months and with faster time to first asymptomatic *P. falciparum* infection indicate that lower child weight is associated with subsequent *P. falciparum* infection. Notably, these findings reveal that even when growth impairment in WAZ does not meet the standard clinical definition of underweight (WAZ < –2.0), milder forms of underweight^66^ can be associated with subsequent health consequences. Studies on the relationship between growth impairment and subsequent malaria have often revealed nonsignificant associations between WAZ and malaria;^47^^,^^74^^–^^76^ however, some studies were conducted in the context of intermittent preventive treatment (IPT) trials, which could complicate interpretations of the relationship between WAZ and malaria, as IPT use has been associated with weight gain.^74^,^77^ Preschool-aged children previously exposed to IPT in Senegal^47^ and who were underweight at baseline exhibited significantly lower cumulative incidence of subsequent malaria (54.1% versus 64.1%; *P* = 0.04) but exhibited no significant association with malaria when underweight status was assessed over time. Similarly, no significant association was observed between being underweight and subsequent malaria among children under 2 years old in Ghana who were exposed to IPT during the study.^74^ Likewise, in a Ugandan cohort study on HIV-infected, HIV-exposed, and uninfected children, both more severe and milder forms of underweight were assessed, and no significant association with subsequent malaria was found, although the results were not stratified by HIV exposure status.^76^

The present study has several limitations. Firstly, in the analysis with WAZ as the outcome, any *P. falciparum* infection that occurred within each quarterly time period was counted as long as it occurred at least 1 day before the WAZ for that time period was measured. This may improperly attribute a WAZ measurement to a too-recent previous *P. falciparum* infection episode and bias the effect estimates of *P. falciparum* infection on WAZ toward null. Additionally, the analysis included only short-term (within 3 months) associations between *P. falciparum* infection and WAZ, which may not have captured the nuanced relationships between these variables; however, analyses of longer-term relationships of the present study’s population have been reported elsewhere.^54^ Some transient asymptomatic *P. falciparum* infections may have been missed because asymptomatic infections were only tested at quarterly visits. Furthermore, data on potentially important confounding variables, including child length, diet, socioeconomic status, and insecticide-treated bednet use, were not available. However, the authors note that unmeasured confounding due to diet is likely limited because of the high prevalence of breastfeeding in this setting for children under 2 years old. All mothers of participants were offered bednets as part of study participation, although their usage was not evaluated. Confounding due to socioeconomic status is likely limited in the study population because the clinic’s catchment area is a relatively homogeneous rural setting with few high-income households. An additional limitation is that the present study was conducted in a setting of high malaria transmission, which could potentially reduce generalizability to other settings. Finally, the analytical sample size of 86 children was small and may have been underpowered to detect significant differences when stratifying by asymptomatic *P. falciparum* infection versus clinical malaria; however, significant associations were identified between WAZ and subsequent *P. falciparum* infection. Given these limitations, the authors cannot infer causality from the associations between *P. falciparum* infection and being mildly to moderately underweight; nevertheless, they believe that the analysis provides a robust first step in establishing the temporality of these common conditions.

## CONCLUSION

In a malaria-endemic setting in rural southern Malawi, using a robust longitudinal analysis, a high incidence of *P. falciparum* infection, including both clinical malaria and asymptomatic *P. falciparum* infections, was found among children under 6 months of age. Children who were mildly to moderately underweight had a higher incidence of subsequent *P. falciparum* infection, and children born mildly to moderately underweight experienced asymptomatic *P. falciparum* infections earlier than those with higher birth WAZ values. These findings can inform malaria interventions and future surveillance decisions in this age group and support targeted interventions to improve child growth and prevent malaria among children with suboptimal weight.

## Supplemental Materials

10.4269/ajtmh.25-0568 Supplemental Materials
