# Supplementary material for: Malaria Incidence, Growth, and their Relationship among a Cohort of Malawian Children
Source: Am J Trop Med Hyg. 2026 Jul 7;115(3):519–27. doi: 10.4269/ajtmh.25-0568 (PMC13435716; doi:10.4269/ajtmh.25-0568)
Supplement: Supplementary file 1 [file tpmd250568.SD1.pdf]

Figure S01: Mean Weight-for-age Z score (WAZ) over time by sex.

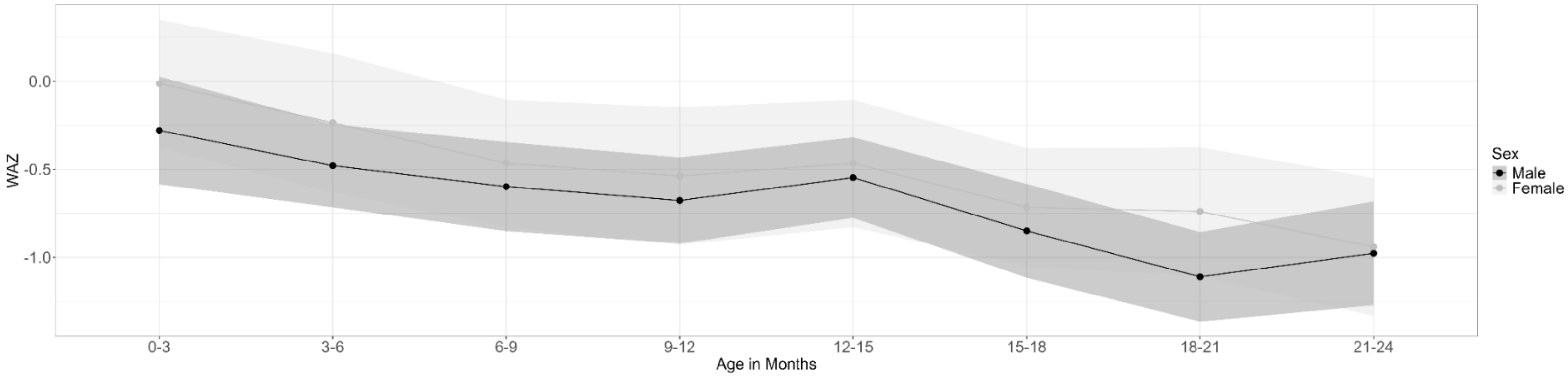

Figure S01: Mean Weight-for-age Z score (WAZ) over time by sex. Mean WAZ and 95% confidence intervals calculated across all observations at each quarterly time period, stratified by whether the observation corresponded to male or female children.

Figure S02: Mean Weight-for-age Z score (WAZ) by number of *P. falciparum* infection episodes experienced by end of follow up.

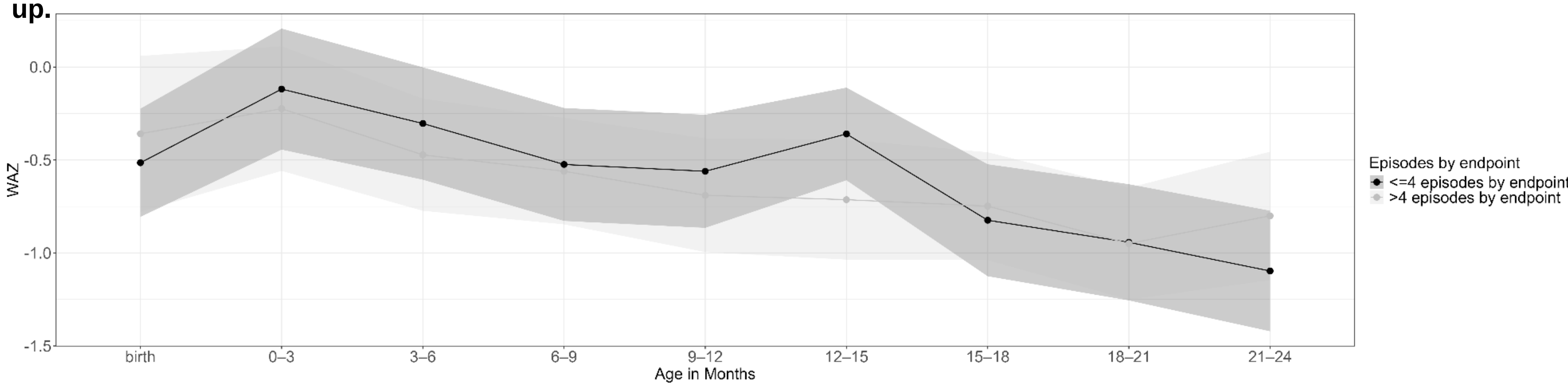

Figure S02: Mean WAZ by number of *P. falciparum* infection episodes experienced by end of follow up. Mean WAZ and 95% CI measured at each quarterly time period from birth to 24 months of age. Children stratified by whether they experienced greater than 4 *P. falciparum* infection episodes by the end of follow-up, which was above the average estimated number of episodes experienced by children in the study [4.36 (3.62, 5.09)]. *P. falciparum* infection includes both asymptomatic *P. falciparum* infection and clinical malaria.

Figure S03: Percentage of children experiencing multiple episodes of *P. falciparum* infection per quarterly time period

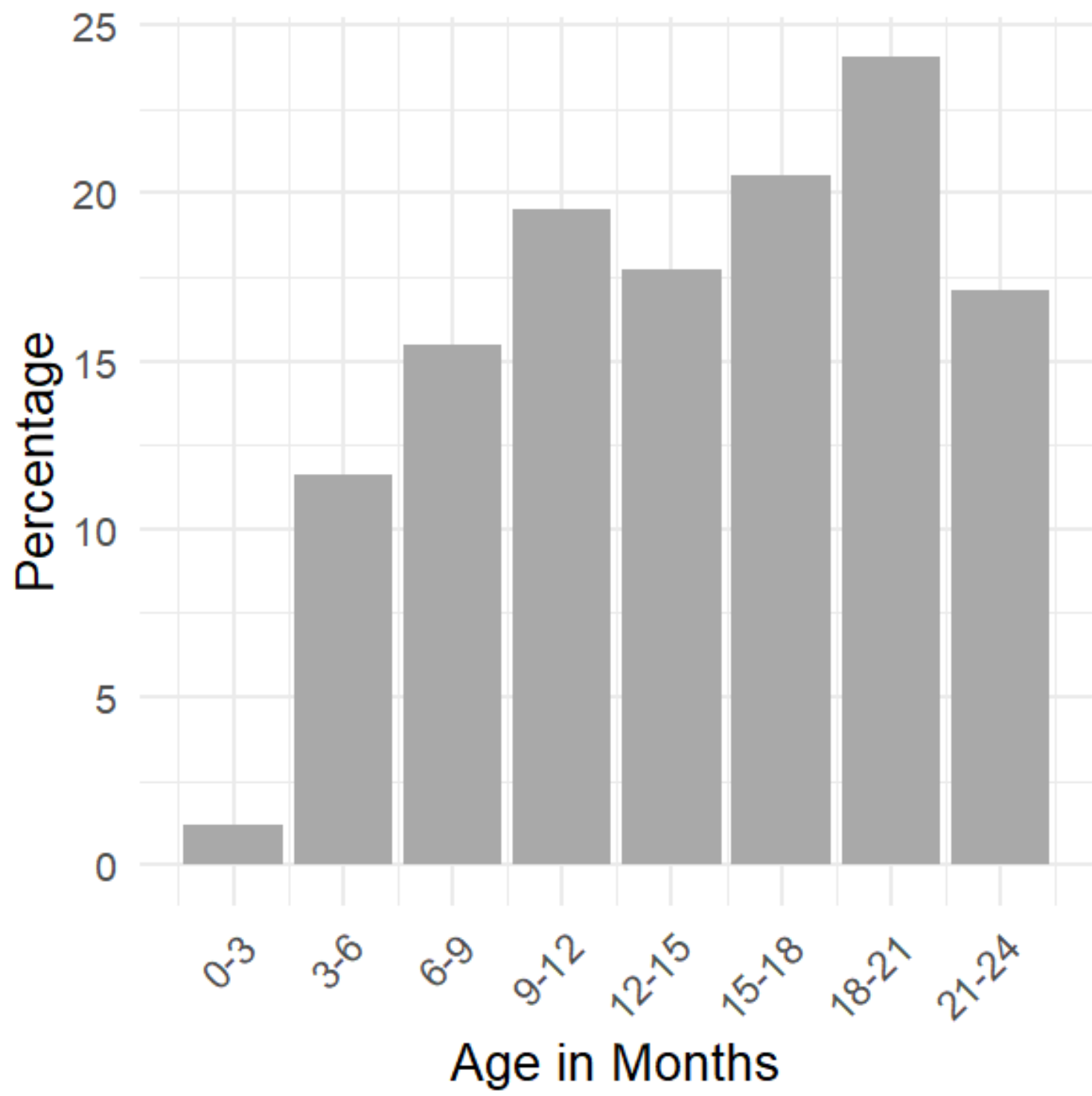

Figure S03: Percentage of children experiencing multiple episodes of *P. falciparum* infection per quarterly time period. The numerator of the percentage represents the number of children who exhibited multiple episodes of *P. falciparum* infection during the quarterly time period. The denominator is the total number of children present during the quarterly time period. *P. falciparum* infection includes both asymptomatic *P. falciparum* infection and clinical malaria.
